# CELER: A 365-Participant Corpus of Eye Movements in L1 and L2 English Reading

**DOI:** 10.1162/opmi_a_00054

**Published:** 2022-07-01

**Authors:** Yevgeni Berzak, Chie Nakamura, Amelia Smith, Emily Weng, Boris Katz, Suzanne Flynn, Roger Levy

**Affiliations:** Technion Israel Institute of Technology, Haifa, Israel; Global Center for Science and Engineering, Waseda University, Tokyo, Japan; Computer Science and Artificial Intelligence Laboratory, Massachusetts Institute of Technology, Cambridge, MA, USA; Linguistics, Massachusetts Institute of Technology, Cambridge, MA, USA; Department of Brain and Cognitive Sciences, Massachusetts Institute of Technology, Cambridge, MA, USA; CBMM: Center for Brains Minds and Machines, Cambridge, MA, USA

**Keywords:** eye movements, corpus, English, L1, L2

## Abstract

We present CELER (**C**orpus of **E**ye Movements in **L**1 and L2 **E**nglish **R**eading), a broad coverage eye-tracking corpus for English. CELER comprises over 320,000 words, and eye-tracking data from 365 participants. Sixty-nine participants are L1 (first language) speakers, and 296 are L2 (second language) speakers from a wide range of English proficiency levels and five different native language backgrounds. As such, CELER has an order of magnitude more L2 participants than any currently available eye movements dataset with L2 readers. Each participant in CELER reads 156 newswire sentences from the *Wall Street Journal* (*WSJ*), in a new experimental design where half of the sentences are shared across participants and half are unique to each participant. We provide analyses that compare L1 and L2 participants with respect to standard reading time measures, as well as the effects of frequency, surprisal, and word length on reading times. These analyses validate the corpus and demonstrate some of its strengths. We envision CELER to enable new types of research on language processing and acquisition, and to facilitate interactions between psycholinguistics and natural language processing (NLP).

## INTRODUCTION

Eye-tracking corpora with naturalistic text, such as the Dundee corpus (Kennedy, 2003; Kennedy et al., 2003) and the Potsdam corpus (Kliegl et al., 2006) have been valuable for the study of human language processing (Demberg & Keller, 2008; Kliegl et al., 2004; Pynte et al., 2008; Smith & Levy, 2013, among others). Despite their utility and availability in several languages, such corpora typically lack L2 (second language) participants, which are needed for studying nonnative language processing and learning. The only publicly available reading dataset with English L2 speakers, the Ghent Eye-Tracking Corpus (GECO; Cop et al., 2017) has only 19 L2 participants, all of whom are university students with the same native language background, Dutch.

To address this gap, we introduce CELER (**C**orpus of **E**ye Movements in **L**1 and L2 **E**nglish **R**eading), an eye-tracking corpus for English with 365 participants, 69 of whom are native speakers of English and 296 are English learners. CELER has more unique text and an order of magnitude more participants than GECO. It has a diverse group of participants, representing a wide range of backgrounds, ages, English proficiency levels, and five native languages: Arabic, Chinese, Japanese, Portuguese and Spanish. Each participant reads 156 newswire sentences from the *Wall Street Journal* (*WSJ*), half of which are shared across all the participants and the remaining half are unique to each participant. This experimental setup aims to enhance the usefulness of CELER by creating two subcorpora, one with a small number of sentences read by many participants, and another with a large number of sentences, each read by a single participant.

CELER has two primary goals. The first goal is to support eye-tracking–based psycholinguistic research on second language processing and acquisition. Thus far, such research has been primarily carried out using controlled textual stimuli, with much of the work focusing on processing of targeted phenomena such as syntactic ambiguities and specific word classes such as cognates (Conklin & Pellicer-Sánchez, 2016; Dussias, 2010; Roberts & Siyanova-Chanturia, 2013). CELER will enable new types of analyses for English L2 reading that require a broad coverage corpus with a large number of participants, such as those performed using existing eye movements corpora with L1 (first language) speakers. It will further facilitate and increase the robustness of comparative studies between L1 and L2 reading in English.

The second goal of CELER is to enhance the interaction between the study of human language processing and natural language processing (NLP). Such connections have been explored, for example, by using NLP language models to study the relation between surprisal and reading times (Goodkind & Bicknell, 2018; Smith & Levy, 2013; Wilcox et al., 2020), and in the integration of eye movement information in NLP systems (Barrett, 2018; Barrett & Hollenstein, 2020; Mathias et al., 2020). Work in these areas relies on the availability of suitable broad-coverage eye-tracking data, and is likely to benefit from extending and diversifying such data in the domain of L2 reading.

## RELATED WORK

CELER is an addition to the existing collection of publicly available broad-coverage eye-tracking corpora for English reading. A widely used such corpus is Dundee (Kennedy, 2003; Kennedy et al., 2003), whose English portion contains 10 subjects reading news editorials presented in paragraphs (51,501 words, 2,368 sentences). The Provo corpus (Luke & Christianson, 2018), has 470 participants reading passages from a diverse range of textual sources (2,689 words, 55 passages, 202 sentences). The UCL (University College London) corpus (Frank et al., 2013) includes 48 participants reading individual sentences taken from novels (205 sentences, 1,931 words). An additional notable resource is the Zurich Cognitive Language Processing Corpus (Hollenstein et al., 2018), which contains simultaneous eye-tracking and EEG recordings from 12 participants (21,629 words, 1107 sentences).

Currently, the only eye movements resource with English L2 participants is GECO (Cop et al., 2017), which contains 14 L1 speakers and 19 L2 speakers whose native language is Dutch. All the participants in GECO are university students. The participants read the novel *The Mysterious Affair at Styles* by Agatha Christie (56,466 words and 4,084 sentences). The L2 group read half of the novel in Dutch and half in English. This corpus has been used for comparing eye-movement measures and frequency effects between L1 and L2 reading (Cop, Drieghe, & Duyck, 2015; Cop, Keuleers et al., 2015).

Although GECO is a first of its kind and a highly valuable resource, CELER introduces several advantages over this dataset. It has more text that is not repeated across participants, and many more L1 and L2 participants. In addition to the number of participants, a crucial advantage of CELER is their diversity. Netherlands is the country with the highest English L2 proficiency worldwide (Education First, 2020); university students whose native language is Dutch, as is the case for GECO, are likely to be at the top of the proficiency range within this already highly proficient group. In CELER, on the other hand, participants are recruited from a wide range of populations, native language backgrounds and English proficiency levels.

Furthermore, all the participants of GECO read the same materials, while CELER also provides a regime in which different readers read different materials. This regime results in a large corpus of text paired with eye movements, which substantially expands the use cases of the dataset. It allows testing generalizability not only across readers but also across text samples, reducing the risk of overfitting to a specific text sample. It further supports the development of real-world applications in which eye movements for the given text are not available from prior readers. We note that CELER also has limitations compared to GECO. Most notably, it uses randomly picked single sentences instead of in-context passages, has less text per participant, and does not contain reading data of the L2 speakers in their native language. Our analyses of CELER include comparisons to GECO, and provide further evidence for the strengths of CELER compared to GECO.

## CORPUS DESCRIPTION

### Participants

CELER comprises 365 participants, of whom 69 are native English speakers and 296 are English L2 speakers from five native language backgrounds: 23 Arabic, 71 Chinese, 71 Japanese, 68 Spanish, and 63 Portuguese. We primarily recruited participants who are not balanced bilinguals. The participants were recruited in the Boston area from a variety of sources: human subjects mailing lists, English L2 schools, language exchange groups, student associations, advertisements on social media, public online and physical message boards, and others. All the participants provided written consent to take part in the experiment, and received monetary compensation for their participation ($20 for L1 participants, and $30 for L2 participants).^1^ We excluded data from participants who did not complete the study, most commonly due to eye-tracker calibration difficulties.

All the participants completed a survey that asked for their native language, age, gender (female, male, or other), level of education (primary, secondary, higher), English age of acquisition (AoA) and time spent in English-speaking countries. We further collected proficiency (beginner, intermediate, advanced, native), AoA, and number of years of language learning and/or usage for any additional language spoken. The L2 participants completed in-lab the Listening and Grammar sections of the Michigan Placement Test (MPT) Form B (henceforth MichiganLG), consisting of 50 questions. Of the 151 L2 participants, 146 have also taken the remaining two sections of the MPT, Vocabulary and Reading Comprehension (henceforth MichiganVR), which consist of 50 additional questions. MPT scores are computed as the number of questions answered correctly. L2 participants also provided scores of the latest standardized English proficiency test taken when available.

**Figure 1.**
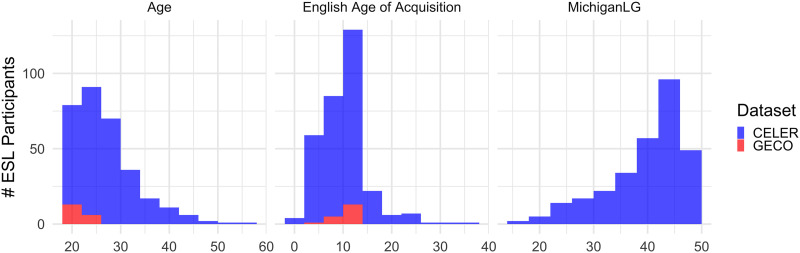
**CELER and GECO English L2 participant distributions for age, English AoA, and CELER MichiganLG scores.** Bar plots are not stacked. CELER = Corpus of Eye Movements in L1 and L2 English Reading; GECO = Ghent Eye-Tracking Corpus; L2 = second language; MichiganLG = Listening and Grammar sections of the Michigan Placement Test.

Table 1 presents participant statistics by native language for CELER and GECO. Figure 1 further depicts the distributions of age, English AoA, and MichiganLG for the L2 participants. We note that in CELER, age, English AoA, and MichiganLG scores are comparable for among the nonnative speaker groups of all five native languages. Further, CELER has substantially wider ranges and better coverage of age and English AoA. The participant survey data and the MPT responses are released as part of the CELER dataset.

**Table 1.** CELER and GECO participant statistics with standard deviation in parentheses.

	**L1**	**# Participants**	**# Female**	**# Male**	**Age**	**English AoA**	**MichiganLG**
**CELER**	Arabic	23	10	13	27.1 (7.5)	8.5 (4.5)	41.5 (6.4)
	Chinese	71	46	25	26.4 (5.8)	9.7 (3.5)	41.0 (6.3)
	Japanese	71	47	24	28.1 (7.8)	10.8 (3.2)	39.2 (6.0)
	Portuguese	63	39	24	28.6 (6.2)	12.5 (5.3)	40.8 (7.2)
	Spanish	68	44	24	27.1 (6.9)	10.7 (6.4)	39.4 (9.2)
	All L2	296	186	110	27.5 (6.8)	10.7 (4.8)	40.2 (7.2)
	English	69	38	28	26.3 (6.7)	0.2 (0.7)	NA
	**All**	365	224	138	27.3 (6.8)	8.7 (6.0)	40.2 (7.2)

**GECO**	Dutch	19	17	2	21.2 (2.1)	11.3 (2.5)	NA
	English	14	8	6	21.8 (5.6)	0 (0)	NA
	**All**	33	25	8	21.5 (3.9)	6.5 (5.9)	NA

*Note*. In CELER (L1 English), one participant indicated “Other” as their gender and two additional participants have not provided gender information. AoA = age of acquisition; CELER = Corpus of Eye Movements in L1 and L2 English Reading; GECO = Ghent Eye-Tracking Corpus; L1 = first language; L2 = second language; MichiganLG = Listening and Grammar sections of the Michigan Placement Test.

### Procedure

The CELER eye-tracking experiment has 157 trials, each consisting of a sentence and subsequent question. The first trial was presented for practice, and is discarded from the data. Seventy-eight of the following sentences belong to a *Shared Text* regime, in which the same sentences are presented to all the participants. The remaining 78 sentences are in the *Individual Text* regime, where each participant is presented with a unique set of sentences. Sentences from the two regimes were interleaved in a fixed order for all participants. The experiment was divided into three blocks, consisting of 52 sentences each. Participants were allowed to take a short break between the blocks. In most cases the duration of the experiment was 45–90 min.

Each sentence was presented on a blank screen as a one-liner. Upon completion of reading each sentence, participants answered a simple yes/no question about its content, and were subsequently informed if they answered the question correctly. Both the sentences and the questions were triggered by a fixation of at least 300 ms on a target (fixation circle for sentences and the letter “Q” for questions) that appeared on a blank screen and was co-located with the beginning of the text in the following screen.

The questions for the Shared Text sentences were composed manually by the experimenters, and test for rudimentary understanding of the sentence content. The questions for the Individual Text sentences were generated automatically, and are of the form “Did the word *X* appear in the sentence?” where *X* is restricted to be a noun, a verb, or an adjective. In both the Individual and Shared Text regimes, half of the correct answers are positive and half are negative.

### Reading Materials

The reading materials of CELER are 28,548 randomly selected newswire sentences from the *WSJ*. To support reading convenience and gaze measurement precision, the maximal sentence length was set to 100 characters. The 78 Shared Text sentences are taken from the test set of the *Wall Street Journal* Penn Treebank (release 2; WSJ-PTB) (Marcus et al., 1993), and have 900 words (11.5 words per sentence). The individual sentences are taken from the training and development sets of the WSJ-PTB, and from the 1987 portion of the BLLIP (Brown Laboratory for Linguistic Information Processing) corpus (Charniak et al., 2000). The Individual Regime materials comprise 28,470 sentences (320,360 words), split into 356 batches of 78 sentences (mean 877.7 words per batch, 11.3 words per sentence).

### Apparatus

The majority of the eye-movement data (253 participants) was recorded using an Eyelink 1000 eyetracker in a desktop mount configuration. The remaining data was collected with an Eyelink 1000 Plus eyetracker in tower mount. In both setups the sampling rate was 1,000 Hz, and eye movements were recorded for the dominant eye of the participant. Further information on the participants, text annotations and the experimental setup is provided in the Supplemental Materials.

## ANALYSES

To validate our corpus and illustrate its strengths, we perform two analyses that reproduce and extend findings from the psycholinguistic literature on eye movements in L1 and L2 reading using CELER and GECO. In the first analysis we follow Cop, Drieghe, and Duyck (2015) and Cop et al. (2017), and benchmark standard eye movement measures in reading. In the second analysis we replicate Whitford and Titone (2012) and Cop, Keuleers et al. (2015), comparing the effect of word frequency on reading times in L1 and L2 speakers, and further extend this comparison to surprisal and word length.

### Analysis 1: Eye Movement Measures

Cop, Drieghe, and Duyck (2015) used GECO to perform a sentence-level analysis of eye movement measures in L1 and L2 reading. They found that L2 reading is characterized by longer sentence reading times (20%), more fixations (21%), shorter saccades (12%), and less word skipping (4.6%), and that the two groups did not differ with respect to regression rates. Cop et al. (2017) further performed word-level analyses obtaining longer L2 reading times for standard word-fixation measures: Single Fixation duration, First Fixation duration, Gaze Duration, and Total Fixation duration. We perform both analyses on the word level for CELER. We also perform these analyses for GECO for the three measures that are available in the public release of the dataset. For each measure, we fit a mixed-effect model that predicts the measure from the English background of the readers (L1 versus L2) with by-subject intercepts. We further examine the interaction of English background with the dataset (CELER versus GECO).

Table 2 presents the results of our analysis. Overall, the differences between L1 and L2 speakers in CELER are consistent in their direction with GECO, including little evidence for a difference in Regression Rate. However, importantly, the differences between L1 and L2 speakers are *substantially larger* in CELER for all the remaining measures. In particular, while in GECO the differences for First Fixation and Gaze Duration are not significant, and for Total Fixation weakly significant, in CELER these differences are highly significant for all three measures. This outcome is likely to reflect the larger diversity of CELER’s L2 participants.

**Table 2.** L1 and L2 means with 95% confidence intervals for eye movement measures.

**Measure**	**CELER**			**GECO**			**English:Dataset**
	**L1**	**L2**	*p*	**L1**	**L2**	*p*	*p*
Single Fixation^1^	213.1_±6.0_	239.3_±3.2_	***	NA	NA	NA	NA
First Fixation^1^	212.3_±6.3_	245.5_±3.5_	***	211.8_±15.0_	220.1_±9.7_	(.)	*
Gaze Duration^1^	245.8_±8.6_	345.0_±8.4_	***	233.9_±18.3_	254.3_±17.1_	(.)	**
Total Fixation^1^	361.5_±21.4_	692.8_±30.7_	***	274.2_±25.9_	310.8_±19.9_	*	**
# of Fixations^2^	1.3_±0.1_	2.6_±0.1_	***	0.8_±0.1_	1.0_±0.1_	***	***
Saccade Length^1^	8.5_±0.4_	6.2_±0.1_	***	NA	NA	NA	NA
Skip Rate^3^	0.36_±0.02_	0.20_±0.01_	***	0.39_±0.03_	0.32_±0.03_	**	**
Regression Rate^3^	0.24_±0.01_	0.26_±0.02_	(.)	NA	NA	NA	NA

*Note*. Single Fixation, First Fixation, Gaze Duration, and Total Fixation times exclude words that were not fixated. The following statistical tests were performed to compare the L1 (first language) and L2 (second language) means in each dataset using the lme4 package in R (Bates et al., 2015), where English ∈ {L1, L2}. (1) lmer(measure ∼ English + (1|participant) (2) glmer(measure ∼ English + (1|participant), family = poisson()) (3) glmer(measure *∼* English + (1|participant), family = binomial()). The last column depicts the significance of the English:Dataset interaction term in the formula: measure ∼ English * Dataset + (1|participant), where Dataset ∈ {CELER, GECO}. *p* values were obtained using the lmerTest package. (.)*p* > .05, *p < .05, **p < .01, ***p < .001. We mark “NA” for measures which are not available in the public release of GECO. CELER = Corpus of Eye Movements in L1 and L2 English Reading; GECO = Ghent Eye-Tracking Corpus.

We further observe that for native speakers, CELER and GECO have similar First Fixation durations, while CELER has longer Gaze Duration and Total Fixation duration, more fixations and lower Skip Rate. This difference is likely to stem at least in part from the different presentation formats and comprehension probing methods, with one-liner sentences and a reading comprehension question after each sentence leading to more rereading. Finally, we note that all the measures are consistent across the Shared and Individual regimes of CELER.

### Analysis 2: The Effect of Frequency, Surprisal, and Word Length on Reading Times

A large body of work in the reading literature has established frequency, predictability, and word length as key factors that affect reading times for native speakers across languages (Kliegl et al., 2004; Rayner et al., 2004; Rayner et al., 2011; Smith & Levy, 2013, among others). Further, Whitford and Titone (2012) have observed a larger frequency effect in English L2 compared to L1. Cop, Keuleers et al. (2015) obtained the same result using GECO.

Here, we replicate the frequency effect result from Whitford and Titone (2012) and Cop, Keuleers et al. (2015) in CELER, and further compare L1 and L2 speakers with respect to surprisal and word length effects. We examine three progressively longer standard fixation measures: First Fixation, Gaze Duration, and Total Fixation. For each measure, we fit a linear mixed-effects model in which the measure is predicted from negative log-frequency, surprisal, and word length of the current and previous words, as well as the interaction of these predictors with the English background of the reader (L1 versus L2). For surprisal, we use Generative Pre-Training 2 (GPT2) (Radford et al., 2019), a state-of-the-art language model, which to our knowledge has not been previously used for analysing L2 reading. In cases where the GPT tokenizer splits a word into multiple tokens, we sum the surprisal values of those tokens. For frequency, we follow Cop, Keuleers et al. (2015) and use SUBTLEX-US (Brysbaert & New, 2009). Word-length values exclude punctuation. Following standard practice, we exclude out-of-vocabulary words, skipped words, words with punctuation, numbers and words that begin or end a trial (sentence for CELER, page for GECO).

The results of our analysis for the current word are presented in Table 3, which also includes GECO. Previous word effects are provided in the Supplemental Materials. First, consistent with the literature, for L1 current word we observe significant main effects for frequency, surprisal, and word length for all three fixation measures in both datasets, with the exception of Total Fixation for frequency in CELER and First Fixation for word length in both datasets. Further, we replicate the interaction between frequency and English background reported by Whitford and Titone (2012) and Cop, Keuleers et al. (2015), obtaining a larger frequency effect for L2 than L1 across all fixation measures in CELER. We note that in GECO, the differences between L1 and L2 frequency effects are not significant in our analysis. New to this work, we also examine the interaction of language background with current word surprisal. Here, we observe an additional important difference between CELER and GECO, whereby we find highly significant interactions for Gaze Duration and Total Fixation in CELER, but no such interactions in GECO. To our knowledge, this result has not been previously reported in the literature, and the finding highlights once more the importance of a large and diverse group of participants for analysing eye movements in L2 reading.

**Table 3.** The effect of current word frequency, surprisal, and word length on reading times in L1 and L2, with 95% confidence intervals.

		**CELER**						**GECO**					
		**FF**		**GD**		**TF**		**FF**		**GD**		**TF**	
	L1 Intercept	214.0_±6.3_		236.9_±7.7_		345.8_±20.4_		210.7_±22.3_		227.2_±26.2_		264.6_±40.8_	
	L2 Intercept	251.3_±3.7_		333.6_±8.0_		684.9_±31.7_		219.7_±19.1_		247.9_±20.8_		301.7_±19.2_	
**Current Word**	L1 Freq	$1.3_{\pm 0.4}^{***}$	]***	$1.9_{\pm 0.5}^{***}$	]***	$3.2_{\pm 1.0}^{***}$	]***	$1.1_{\pm 0.3}^{***}$	]^(.)^	$1.4_{\pm 0.5}^{***}$	]^(.)^	$1.4_{\pm 1.0}^{**}$	]^(.)^
	L2 Freq	$3.2_{\pm 0.2}^{***}$		$8.1_{\pm 0.6}^{***}$		$22.7_{\pm 1.9}^{***}$		$1.3_{\pm 0.4}^{***}$		$1.6_{\pm 0.6}^{***}$		$2.3_{\pm 0.9}^{***}$	
	L1 Surp	$0.6_{\pm 0.3}^{***}$	]^(.)^	$1.9_{\pm 0.7}^{***}$	]***	$6.7_{\pm 1.0}^{***}$	]***	$0.6_{\pm 0.1}^{***}$	]^(.)^	$1.1_{\pm 0.2}^{***}$	]^(.)^	$3.0_{\pm 0.8}^{***}$	]^(.)^
	L2 Surp	$0.6_{\pm 0.1}^{***}$		$2.9_{\pm 0.3}^{***}$		$13.7_{\pm 1.0}^{***}$		$0.5_{\pm 0.3}^{***}$		$1.1_{\pm 0.4}^{***}$		$3.0_{\pm 0.6}^{***}$	
	L1 Len	$-0.8_{\pm 0.9}^{\left(.\right)}$	]^(.)^	$4.3_{\pm 1.5}^{***}$	]***	$14.5_{\pm 2.5}^{***}$	]***	$-0.2_{\pm 0.5}^{\left(.\right)}$	]*	$4.5_{\pm 1.9}^{***}$	]*	$8.6_{\pm 2.4}^{***}$	]*
	L2 Len	$-1.2_{\pm 0.4}^{***}$		$19.6_{\pm 1.8}^{***}$		$42.7_{\pm 3.9}^{***}$		$-0.6_{\pm 0.6}^{\left(.\right)}$		$9.2_{\pm 3.1}^{***}$		$14.5_{\pm 2.9}^{***}$	

*Note*. RT ∼ Freq + Freq_prev + Surp + Surp_prev + Len + Len_prev + (Freq + Freq_prev + Surp + Surp_prev + Len + Len_prev|participant). Interactions between English background and word properties tested by: RT ∼ English * Freq + English * Freq_prev + English * Surp + English * Surp_prev + English * Len + English * Len_prev + (Freq + Freq_prev + Surp + Surp_prev + Len + Len_prev|participant). Adding a (Surp|Word_Type) random effect resulted in model convergence issues with GECO and was therefore not included. The omission of this random effect did not lead to qualitative changes in the model coefficient estimates for CELER. All predictors are centered. (.)*p* > .05, *p < .05, **p < .01, ***p < .001. Tests performed using the MixedModels library in Julia (Bezanson et al., 2017). CELER = Corpus of Eye Movements in L1 and L2 English Reading; FF = First Fixation; GD = Gaze Duration; L1 = first language; L2 = second language; TF = Total Fixation; GECO = Ghent Eye-Tracking Corpus.

## USES OF THE CORPUS

A subset of CELER (CELER v1) was previously used in two studies, Berzak et al. (2017) and Berzak et al. (2018). Berzak et al. (2017) used the L2 part of the data, and capitalized on the four native languages of the L2 participants in CELER v1 to demonstrate that the native language of L2 speakers can be decoded from eye-movement features during reading. Berzak et al. (2018) also utilized the native portion of the corpus, as well as the MPT and TOEFL scores of the L2 participants to develop an eye-tracking–based method for estimating the English proficiency of L2 learners. Overall, these studies demonstrate the potential of the corpus for combining eye movements with NLP and supporting new research directions on second language acquisition.

## CONCLUSION

We presented CELER, a broad coverage eye-tracking corpus for English with L1 and L2 speakers. We envision that the corpus will support a wide range of research in psycholinguistics and NLP, contribute to cross fertilization between these and adjacent fields, and facilitate advancements in our understanding of human language processing. We also hope that CELER will inform future efforts for collecting large-scale eye-tracking data for both native and learner participant populations.

## FUNDING INFORMATION

BK and YB: the Center for Brains, Minds, and Machines, NSF STC award (https://dx.doi.org/10.13039/100000001), Award ID: CCF-1231216. RL and YB: RI: Small: Computational analysis of eye movements in reading: reader characteristics, cognitive state, and natural language processing, National Science Foundation (https://dx.doi.org/10.13039/100000001), Award ID: 1815529. RL: MIT-IBM Research Lab. RL: MIT Quest for Intelligence. CN: JSPS KAKENHI Grant Number 21KK0006.

## AUTHOR CONTRIBUTIONS

YB: Conceptualization, Data Curation, Formal Analysis, Investigation, Methodology, Project Administration, Supervision, Validation, Visualization, Writing - Original Draft, Writing - Review and Editing. CN: Conceptualization, Investigation, Methodology, Writing - Review and Editing. AS: Investigation, Project Administration. EW: Data Curation, Investigation. BK: Conceptualization, Supervision, Writing - Review and Editing. SF: Conceptualization, Methodology, Supervision, Writing - Review and Editing. RL: Conceptualization, Formal Analysis, Methodology, Supervision, Writing - Original Draft, Writing - Review and Editing.

## Note

^1^ Under MIT IRB protocols 1502006957 and 1605559077.

## Supplemental Materials

https://github.com/berzak/celer
