# Supplementary material for: CELER: A 365-Participant Corpus of Eye Movements in L1 and L2 English Reading
Source: Open Mind (Camb). 2022 Jul 1;6:41–50. doi: 10.1162/opmi_a_00054 (PMC9692049; doi:10.1162/opmi_a_00054)
Supplement: Supplementary file 1 [file opmi-06-41-s001.pdf]

== D R A F T March 12, 2022 (Received) ==

Journal: **OPEN MIND**

CELER: A 365-Participant Corpus of Eye Movements in L1 and L2 English Reading

Yevgeni Berzak[1], Chie Nakamura[2], Amelia Smith[3], Emily Weng[3], Boris Katz[3], Suzanne Flynn[4] and Roger Levy[5]

[1]Technion Israel Institute of Technology, Haifa, Israel

[2]Waseda University, Tokyo, Japan

[3]CSAIL, Massachusetts Institute of Technology, Cambridge, USA

[4]Linguistics, Massachusetts Institute of Technology, Cambridge, USA

[5]BCS, Massachusetts Institute of Technology, Cambridge, USA

SUPPLEMENTARY INFORMATION

Dataset Versions

CELER has two major versions. The first version, v1, contains 182 participants (37 L1, 145 L2 from four native language backgrounds: Chinese, Japanese, Portuguese and Spanish). The current version, v2, has 183 additional participants (32 L1, 151 L2 from the same native languages as in v1, as well as Arabic) with a total of 365 participants. In v2, a special effort was made to increase the portion of L2 participants with intermediate and low English proficiency levels, as well as L1 speakers from communities outside the university campus. The individual sentences of CELER are taken from the WSJ-PTB in v1, and from the BLLIP corpus (Charniak et al., 2000) for participants new to v2. The MichiganVR was taken only by participants new to v2.

Participant Native Languages

359 of the participants indicated a single language as native. Of the remaining 6 participants 3 are native speakers of Spanish and Catalan, 1 Chinese and Tibetian, 1 Arabic and French and 1 English and Yiddish. We also note that one of the participants counted as L1 Spanish, is a native speaker of Catalan and advanced speaker of Spanish.

Corresponding author: Yevgeni Berzak, berzak@technion.ac.il

Copyright ©2022 The Author(s). Published by MIT Press. All Rights Reserved.

For most L2 participants, information is available on native language country for Arabic, Spanish and Portuguese, and native language variety for Chinese:

- 16 Arabic: 5 Saudi Arabia, 1 Egypt, 1 Iraq, 1 Jordan, 1 Kuwait, 1 Lebanon, 1 Morocco, 1 Oman, 1 Sudan, 1 Syria, Tunisia, 1 bilingual Egypt and UAE.
- 56 Chinese: 52 Mandarin, 1 Cantonese, 3 bilingual Mandarin and Cantonese.
- 42 Portuguese: 38 Brazil, 4 Portugal.
- 38 Spanish: 14 Spain, 7 Colombia, 6 Mexico, 4 Venezuela, 3 Peru, 1 Argentina, 1 Chile, 1 El Salvador, 1 Puerto Rico.

Text Annotations

All the sentences are provided with their syntactic annotations from the Penn Treebank (manual) and BLLIP (automatic). We further use the Universal Dependency Treebank (UDT) (McDonald et al., 2013) conversion tool to generate Google universal POS tags (Petrov, Das, & McDonald, 2012) and dependency trees. Additionally, we provide three word properties for each word w_i: negative log-frequency (negative log-unigram probability): $-\log_2 p(w_i)$, surprisal: $-\log_2 p(w_i|w_1, ..., w_{i-1})$ and word length. Frequency values are available in three variants, from BLLIP and SUBTLEX-US (Brysbaert & New, 2009), and Kaggle Web word frequency list. Surprisal values are obtained using the state-of-the-art language model GPT2 (Radford et al., 2019), as well as LSTM and ngram models trained on BLLIP. When using BLLIP for extracting word frequencies and training language models we exclude sentences that appear in CELER. Further details on the LSTM and ngram models are provided in the dataset documentation.

Apparatus and Text Presentation

We used a 27inch monitor with 2560x1440px resolution. For participants in v1, the presentation area of the experiment was 45.3x34cm rescaled to 1024x768px resolution. For participants new to v2 we used the native resolution of the monitor, with the same physical positioning of the text and text size. Participants' eyes were approximately 78cm from the center of the monitor. Participants' head was fixed using a chinrest.

The text was presented on the central horizontal line of the monitor in 29pt (23px in v1, 43px in v2) Times New Roman font. In our setup, this corresponds to approximately 0.36 degrees average lower case

letter width, and 0.49 degrees average upper case letter width. We chose Times New Roman, a non-monospace font, as this font is more commonly used compared to monospace fonts. Times New Roman is also more compact compared to monospace fonts, allowing to fit longer sentences in one line.

We use 3H line calibration with point repetition on the central horizontal line. At least three calibrations are performed during the experiment, one at the beginning of each experimental section. We also recalibrate upon failure to produce a 300ms fixation on any fixation target preceding a sentence or (and in v1 a "Q" target preceding a question) within 4 seconds after its appearance. Experimenters were instructed to repeat calibration if the validation error was above 0.2 degrees. Further details on the experimental setup are presented in the documentation of the released data.

Analysis 2: Previous Word

	CELER						GECO					
**Previous Word**	**FF**		**GD**		**TF**		**FF**		**GD**		**TF**	
L1 Freq	$1.0^{***}_{\pm 0.5}$	]$^{(.)}$	$1.6^{***}_{\pm 0.6}$	]$^{(.)}$	$2.2^{***}_{\pm 1.0}$	]$^{(.)}$	$-0.4^{***}_{\pm 0.2}$	]$^{(.)}$	$-0.3^{(.)}_{\pm 0.2}$	]$^{(.)}$	$-0.3^{(.)}_{\pm 0.5}$	]$^{(.)}$
L2 Freq	$1.4^{***}_{\pm 0.2}$		$1.8^{***}_{\pm 0.3}$		$2.3^{***}_{\pm 1.0}$		$0.0^{(.)}_{\pm 0.4}$		$0.0^{(.)}_{\pm 0.5}$		$0.2^{(.)}_{\pm 0.6}$	
L1 Surp	$-0.4^{**}_{\pm 0.3}$	]$^{(.)}$	$-0.1^{(.)}_{\pm 0.4}$	]$^{(.)}$	$1.9^{***}_{\pm 0.7}$	]***	$0.6^{***}_{\pm 0.2}$	]$^{(.)}$	$1.0^{***}_{\pm 0.3}$	]$^{(.)}$	$2.1^{***}_{\pm 0.6}$	]$^{(.)}$
L2 Surp	$-0.2^{**}_{\pm 0.1}$		$0.3^{**}_{\pm 0.2}$		$7.8^{***}_{\pm 0.8}$		$0.7^{***}_{\pm 0.2}$		$1.1^{***}_{\pm 0.3}$		$2.4^{***}_{\pm 0.4}$	
L1 Len	$-0.8^{*}_{\pm 0.8}$	]$^{(.)}$	$-2.3^{***}_{\pm 0.9}$	]$^{(.)}$	$-6.8^{***}_{\pm 1.6}$	]***	$-1.3^{***}_{\pm 0.5}$	]*	$-2.3^{***}_{\pm 0.7}$	]*	$-4.7^{***}_{\pm 2.1}$	]*
L2 Len	$-1.4^{***}_{\pm 0.3}$		$-3.2^{***}_{\pm 0.6}$		$-18.3^{***}_{\pm 1.5}$		$-2.0^{***}_{\pm 0.5}$		$-3.9^{***}_{\pm 1.1}$		$-7.7^{***}_{\pm 1.0}$	

Table 1. The effect of previous word frequency, surprisal and word length on reading times in L1 and L2, with 95% confidence intervals: RT $\sim$ Freq + Freq_prev + Surp + Surp_prev + Len + Len_prev + (Freq + Freq_prev + Surp + Surp_prev + Len + Len_prev|participant). Interactions between English background and word properties tested by: RT $\sim$ English $*$ Freq + English $*$ Freq_prev + English $*$ Surp + English $*$ Surp_prev + English $*$ Len + English $*$ Len_prev + (Freq + Freq_prev + Surp + Surp_prev + Len + Len_prev|participant). All predictors are centered. '***' $p < 0.001$, '**' $p < 0.01$. '*' $p < 0.05$, '(.)' $p > 0.05$. Tests performed using the MixedModels library in Julia.

In line with prior literature (Pollatsek, Juhasz, Reichle, Machacek, & Rayner, 2008), both CELER and GECO show reverse spillover effects for word length in L1, and a consistent tendency for larger word

length effects in L2.[1] We further observe significant frequency effects in CELER L1 and L2 but not in GECO, and find that the differences in frequency effects between the datasets are significant for all measures in both language background groups ($p < 0.01$), except for Total Fixation in L2.[2] On the other hand, differently from CELER, GECO exhibits clear previous word surprisal effects in both L1 and L2 for First Fixation and Gaze Duration with significant differences between the effects across the datasets ($p < 0.01$).[3] Both datasets show significant previous word Total Fixation effects for surprisal in L1 and L2, where in CELER we further observe a significant interaction between language background and surprisal which is not present in GECO.[4] We also note a nearly significant interaction between language background and previous word frequency on First Fixation duration ($p = 0.06$).

REFERENCES

Brysbaert, M., & New, B. (2009). Moving beyond kučera and francis: A critical evaluation of current word frequency norms and the introduction of a new and improved word frequency measure for american English. *Behavior research methods*, *41*(4), 977–990.

Charniak, E., Blaheta, D., Ge, N., Hall, K., Hale, J., & Johnson, M. (2000). BLLIP 1987-89 wsj corpus release 1. *Linguistic Data Consortium, Philadelphia*, *36*.

McDonald, R. T., Nivre, J., Quirmbach-Brundage, Y., Goldberg, Y., Das, D., Ganchev, K., ... others (2013). Universal dependency annotation for multilingual parsing. In *ACL* (pp. 92–97).

Petrov, S., Das, D., & McDonald, R. (2012). A universal part-of-speech tagset. In *LREC*.

Pollatsek, A., Juhasz, B. J., Reichle, E. D., Machacek, D., & Rayner, K. (2008). Immediate and delayed effects of word frequency and word length on eye movements in reading: A reversed delayed effect of word length. *Journal of experimental psychology: human perception and performance*, *34*(3), 726.

[1] Such effects can be explained as follows: when word n-1 is longer, the reader spends more time fixating on it. This leads to more parafoveal preview of word n and therefore shortens its reading time.

[2] Freq_prev:Dataset term in the following formula applied separately for the L1 and L2 populations: $\text{RT} \sim \text{Dataset} * \text{Freq} + \text{Dataset} * \text{Freq\_prev} + \text{Dataset} * \text{Surp} + \text{Dataset} * \text{Surp\_prev} + \text{Dataset} * \text{Len} + \text{Dataset} * \text{Len\_prev} + (\text{Freq} + \text{Freq\_prev} + \text{Surp} + \text{Surp\_prev} + \text{Len} + \text{Len\_prev} | \text{participant})$.

[3] Surp_prev:Dataset interaction in the formula of footnote 6.

[4] The interaction Dataset:English:Surp_prev is significant with $p = 0.0102$ in $\text{RT} \sim \text{English} * \text{Dataset} * \text{Freq} + \text{English} * \text{Dataset} * \text{Freq\_prev} + \text{English} * \text{Dataset} * \text{Surp} + \text{English} * \text{Dataset} * \text{Surp\_prev} + \text{English} * \text{Dataset} * \text{Len} + \text{English} * \text{Dataset} * \text{Len\_prev} + (\text{Freq} + \text{Freq\_prev} + \text{Surp} + \text{Surp\_prev} + \text{Len} + \text{Len\_prev} | \text{participant})$.

Radford, A., Wu, J., Child, R., Luan, D., Amodei, D., & Sutskever, I. (2019). Language models are unsupervised multitask learners.
